# The pre-Argo ocean reanalyses may be seriously affected by the spatial coverage of moored buoys

**DOI:** 10.1038/srep46685

**Published:** 2017-04-21

**Authors:** S. Sivareddy, Arya Paul, Travis Sluka, M. Ravichandran, Eugenia Kalnay

**Affiliations:** 1 ESSO-Indian National Centre for Ocean Information Services, Ministry of Earth Sciences, Pragathi Nagar, Hyderabad, 500090, India; 2 Department of Atmospheric and Oceanic Sciences, University of Maryland, College Park, Maryland, USA; 3 ESSO-National Centre for Antarctic and Ocean Research, Ministry of Earth Sciences, Headland Sada, Vasco-da-Gama, Goa 403804, India

## Abstract

Assimilation methods, meant to constrain divergence of model trajectory from reality using observations, do not exactly satisfy the physical laws governing the model state variables. This allows mismatches in the analysis in the vicinity of observation locations where the effect of assimilation is most prominent. These mismatches are usually mitigated either by the model dynamics in between the analysis cycles and/or by assimilation at the next analysis cycle. However, if the observations coverage is limited in space, as it was in the ocean before the Argo era, these mechanisms may be insufficient to dampen the mismatches, which we call shocks, and they may remain and grow. Here we show through controlled experiments, using real and simulated observations in two different ocean models and assimilation systems, that such shocks are generated in the ocean at the lateral boundaries of the moored buoy network. They thrive and propagate westward as Rossby waves along these boundaries. However, these shocks are essentially eliminated by the assimilation of near-homogenous global Argo distribution. These findings question the fidelity of ocean reanalysis products in the pre-Argo era. For example, a reanalysis that ignores Argo floats and assimilates only moored buoys, wrongly represents 2008 as a negative Indian Ocean Dipole year.

Observations are periodically used to arrest the drifting away of oceanic models from reality using data assimilation. Use of data assimilation has not only improved ocean state forecasts on daily timescales but is also known to have produced better ocean reanalyses (historical ocean states) based on which multiple climate change interpretations^1^,^2^,^3^,^4^,^5^,^6^,^7^ have been made. There are a number of data assimilation methods applied to numerical ocean models ranging in varying levels of complexity -from simple interpolation methods^8^ to methods based on Bayesian Statistics^9^,^10^,^11^,^12^,^13^. Most present day operational centers use state-of-the-art methods like 3DVar, 4DVar or some variant of Ensemble Kalman Filter. Typical assimilation cycles, in simple terms, are as follows - the numerical model evolves for a short period of time and generates a background model state; all the available observations are collected and used to correct the model state through assimilation; this process generates an analysis; the model evolves it again using this analysis as the updated initial condition, and this cycle continues. However, the analysis doesn’t explicitly satisfy the dynamical equations of the model, thereby giving rise to an unbalanced initial state for the next cycle. These perturbations, arising from upsetting the balance, propagate as transient waves. Given sufficient time, these spurious waves are generally dissipated by the model dynamics itself. They can also be mitigated by the advent of new observations in the next analysis cycle. However, if these mechanisms are weak or absent, these perturbations can become stronger with each analysis cycle, propagate and disturb the ocean state thereby counteracting the purpose of assimilation, which is to make the model evolution more realistic by forcing it to remain close to the observations. It is thus important to look into systems where there may be a detrimental impact of the assimilation.

It is interesting to consider a type of systems where such rogue waves may thrive. For example, assimilation of observations from fixed-location instruments at regular temporal intervals can persistently perturb the ocean especially at and around those observation locations and thus trigger these waves. If the time interval between two assimilation cycles is smaller than the model dispersion and dissipation time scales, these effects won’t be sufficiently damped before the next analysis cycle and they may potentially thrive. A possible type of observations that may cause such spurious effects are moored buoys in the ocean which are anchored at fixed locations^14^. They typically provide time series of temperature and salinity observations from the surface to 500 m depth. The moored buoys are confined in the Pacific Ocean within 10°*S*-10°*N* thereby forming a distinct boundary between regions in the ocean influenced by the assimilation, and regions unaffected during each analysis cycle. This mismatch at the latitudinal interface can lead to undesirable boundary effects. In order to test the existence of such rogue waves in ocean analysis, we conduct Observation System Experiments (OSEs) meant to assess the impact of real observations in data assimilation. Later we repeat these experiments with simulated observations and a different model and method of data assimilation to confirm the robustness of our results.

## Observation System Experiments

A set of OSEs is conducted on a global ocean data assimilation system INCOIS-GODAS^12^,^15^ that comprises of the numerical ocean model MOM4.0^16^ and 3D-VAR assimilation scheme^17^ which can assimilate temperature and salinity profiles. The system simulates ocean state in the Pacific and Indian Ocean reasonably well especially at monthly to inter-annual time scales. Inter-comparisons between analyses from various real ocean data assimilation systems^18^ indicate that the errors are large in the Atlantic Ocean in all ocean data assimilation systems. This is true for INCOIS-GODAS as well^12^. Hence results from OSEs are highlighted only in the Pacific and the Indian Ocean. These OSEs involve assimilation of moored buoys and Argo. Argo is a global network of free moving floats in the ocean that typically surface every 10 days and relay ocean temperature and salinity measurements from the surface to 2000 m^19^. While moored buoys operate at its deployed location, Argo floats drift and measure temperature and salinity at different locations in ocean. The following experiments are conducted under OSE for the period 2004–2011:

*FR* Free run experiment where no observations are assimilated.

*MB* experiment where *in-situ* temperature and salinity observations from Moored Buoys are assimilated.

*AR* experiment where *in-situ* temperature and salinity observations from Argo floats are assimilated.

*AR*+*MB* experiment where *in-situ* temperature and salinity observations from both Argo floats and moored buoys are assimilated.

More details about the configuration of INCOIS-GODAS can be found in the supplementary information. We use a subscript “R” in the names of these experiments to indicate usage of real observations.

**Figure 1:**
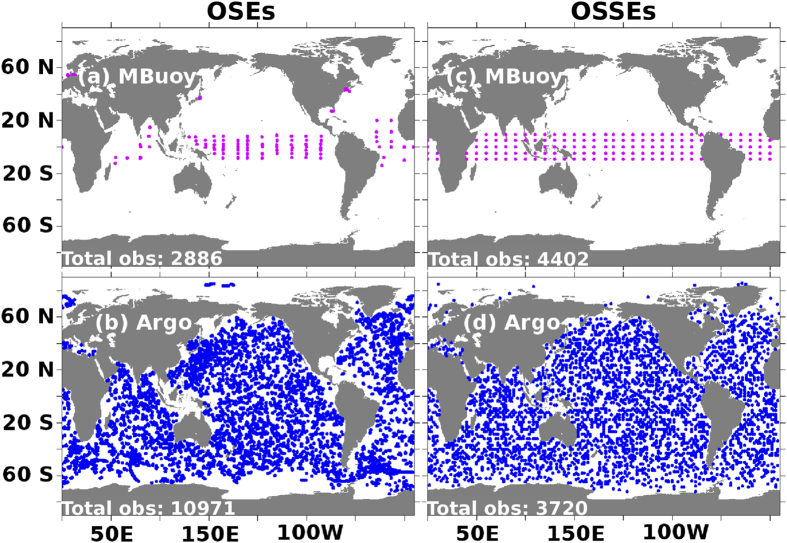
Panel (a,b) show observation coverage of real moored buoys and real Argo floats during January, 2009 used in OSEs. Panel (c,d) observation coverage of simulated moored buoys and simulated Argo floats used in OSSEs during January in the second year of assimilation. The total number of observations are also indicated in the corresponding panel. Any observation beyond 60°S-60°N is not assimilated. Images are generated and processed with the help of FERRET-V6.3 (www.ferret.noaa.gov/Ferret) and GIMP-V2.8 (www.gimp.org) respectively.

Bias correction in XBT data has been shown to have larger impact on the estimations^20^ of various parameters such as inter-decadal variabilities of ocean heat content^21^, decadal variabilities of thermo-steric sea level^22^ etc. Hence in the OSE study, we employed temperature and salinity profiles of “XBT corrected” Ensemble quality controlled version-2a (EN2a^22^,^23^) data set from the UK Met Office. This EN2a data set is chosen due to its rich collection of *in-situ* temperature and salinity profiles (acquired from various projects, i.e. WOD05, GTSPP, Argo, and ASBO), subjected to rigorous quality checks^23^. We have considered delayed mode and high quality profiles - inferred from position QC and profile QC, but discarded profiles with large vertical data gaps. Since the present study is primarily based on observations from moored buoy and Argo networks, in Fig. 1a and 1b, we highlight the typical observation coverage over a month from the moored buoy and Argo respectively. Each day about 100 profiles from moored buoys and about 350 profiles from Argo are assimilated on average. Observations falling outside the latitude band 60°N to 60°S are not assimilated.

The assimilation of temperature and salinity observations from (real) moored buoy network (*MB*_*R*_ experiment) is found, as expected, to improve the mean and the variability of temperature and salinity analysis within the region of observational coverage compared to their corresponding free run. Assimilation of temperature and salinity from moored buoys also induces marked improvements in Sea Surface Height (SSH) thereby indicating the ability of INCOIS-GODAS produce useful data assimilation. For more discussion on its quality please refer to the supplementary information.

**Figure 2:**
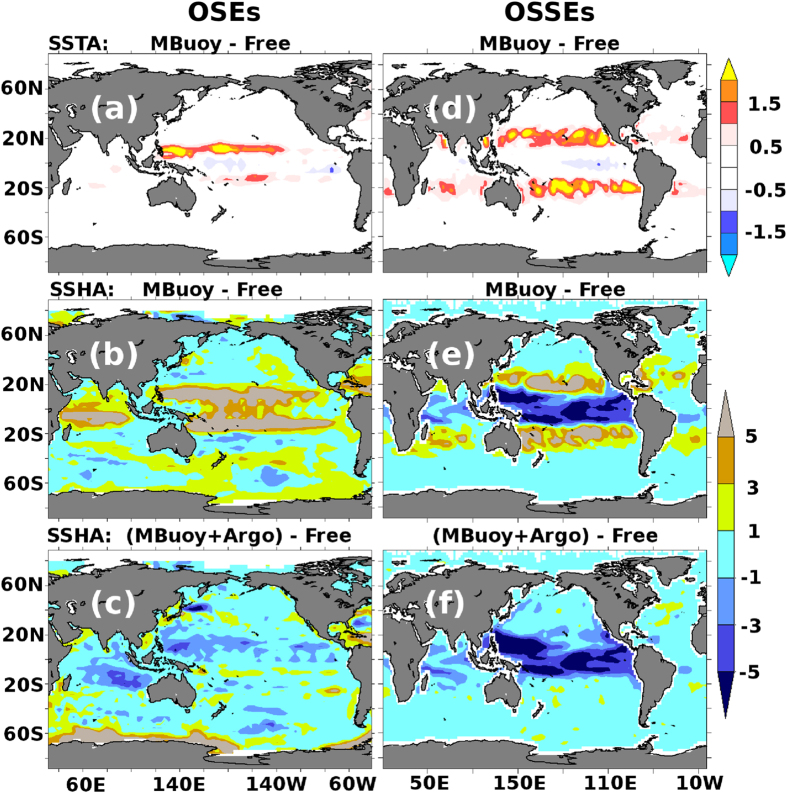
Root-mean-square error differences between free and assimilation experiments in OSEs (**a,b,c**) and OSSEs (**d,e,f**). Panels (a,d) show root-mean-squared error differences in SSTA (°C) between MB and FR. Panels (b,e) show root-mean-squared error differences in SSHA (cm) between MB and FR. Similarly, panels (c,f) show root-mean-squared error differences in SSHA (cm) between MB+AR and FR. In the figure positive (negative) values indicate degradation (improvements) from the assimilation experiment with respect to free experiment. Root-mean-squared-error for each OSSEs is computed with respect to *Nature* run, whereas in OSEs it is computed with respect to satellite based SST observations of TMIAMSRE and satellite altimeter based SSHA global maps from AVISO. Images are generated and processed with the help of FERRET-V6.3 (www.ferret.noaa.gov/Ferret) and GIMP-V2.8 (www.gimp.org) respectively.

The free model run (*FR*_*R*_) presents large deviations (~1 °C) in sea surface temperature anomaly (SSTA) on the eastern and western side of the Equator in the Pacific Ocean when compared to the TMIAMSRE^24^ (merged product based on TMI and AMSRE satellite SST measurements) observations (figure not shown). These differences are largely eliminated once moored buoys are assimilated (*MB*_*R*_ experiment). This is illustrated in Fig. 2a where difference in RMSE between the *MB*_*R*_ experiment and *FR*_*R*_ run is plotted. Assimilation, however, introduces errors with large spatial extent along the latitudinal boundaries of the moored-buoy coverage, i.e., at 10°*S* and 10°*N*. This spatial degradation in the SST analysis also impacts other variables like Sea Surface Height Anomaly (SSHA) (Fig. 2b). These large errors at the edges of moored buoy network appear to be present in other assimilation systems as well. For example, results from OSEs based on NCEP-GODAS and GFDL-ECDA^25^, where temperature and salinity from moored buoys are assimilated, also indicate degradations in ocean state variables in the tropical Pacific Ocean along the latitudes outside the moored buoy observational coverage. Even though the authors did not explicitly mention the degradations, they are evident when Fig. 12e,g in ref. 25 are compared. Since all the above systems including ours use a version of MOM as the numerical model, these degradations could be model specific. Another possibility is that fewer number of salinity observations compared to temperature from the moored buoys in the Pacific Ocean might have generated imbalances in the ocean state^26^.

In order to explore these possibilities, we resort to Observation System Simulation Experiments (OSSEs) that involve assimilation of simulated observations. Unlike OSEs that investigate and analyze the impact of real observations, OSSEs are designed to carry out controlled experiments with simulated observations and gauge their impact. OSSEs necessitate a controlled model run called “Nature” run generating states that are the “truth” of the system. Simulated observations are generated from the Nature run by adding random “observational” errors. The model used for forecasting the Nature run is then deliberately made imperfect by tweaking some aspects of the model, and perturbing the initial conditions. However, both the Nature run and the imperfect forecast model should mimic reality reasonably well at least at the scales of interest. Simulated observations are assimilated to this imperfect model and the analysis so generated is compared with the free imperfect model run to assess improvements/degradations brought by the data assimilation compared to the true system (Nature run). OSSEs offer several advantages compared to OSEs - e.g., knowledge of truth and complete control in generating observations at intended spatio-temporal scales.

## Observation System Simulation Experiments

In the OSSEs, we use a different assimilation system (SPEEDY-NEMO-LETKF^27^) comprising of a different numerical ocean model, NEMO^28^ and a different assimilation scheme, LETKF^29^ in order to test if the errors are an offshoot of the model and/or the assimilation scheme characteristics. Simulated temperature and salinity observations, spatially and temporally similar to that of the real tropical moored buoy and global Argo observation network, were generated by adding random errors to the Nature run. The existence of errors in OSSEs similar to those in OSEs would negate the possibility of these errors being model and/or assimilation scheme specific or due to an imbalance in the number of salinity and temperature observations.

After a 20 year spin-up of the coupled ocean-atmospheric model SPEEDY-NEMO^27^,^30^, we evolve it for 22 years and consider these last 22 years as the Nature run (truth). Sea-surface temperature and sea-surface salinity are relaxed to their respective monthly climatologies^31^. The results capture most of the prominent ocean variabilities at scales larger than intra-seasonal scales^27^. The ocean model is then made “imperfect” by turning off the relaxations and perturbing the initial conditions. We then execute experiments similar to those of OSEs. We use subscript “S” that indicates use of simulated observations to distinguish experiments under OSSEs from OSEs. To generate moored-buoy-like observations for *MB*_*S*_experiment, temperature and salinity are sampled every 24 hours at spatial intervals of 10° in the zonal and 5° in the meridional direction and at alternate ocean model depths starting from surface to 500 *m* in the vertical direction. Unless otherwise specified, simulated moored buoy observations are designed to be, like the real moored buoys, confined within 10°*S*–10°*N*. Argo-like observations (*AR*_*S*_) are generated daily by sampling temperature and salinity profiles from surface to 2000 m at alternate model depths at 120 random spatial locations. Fewer Argo-like observations than the real number are sufficient to cover the globe over a month in OSSEs and are enough to constrain the drifting of the model trajectory in NEMO, since its resolution is four times coarser than MOM’s. Figure 1c and d illustrate the spatial coverage of simulated observations from moored buoy and Argo respectively over a month in OSSEs. For more details about the set-up and experiments, please refer to the supplementary information.

Simulations of SSTA from simulated Free Run (FR_S_) show large errors in the eastern parts of the Equatorial Pacific Ocean when compared to the Nature run. The SSHA simulations are also fraught with large errors in the Equatorial Pacific Ocean (figure not shown). These errors are largely mitigated with the assimilation of simulated moored buoy observations. In contrast to SSHA results of OSSEs, OSEs show degradations within the moored buoy coverage area (shown in Fig. 2b). A detailed discussion on these contrasting SSHA results between OSEs and OSSEs is provided in supplementary section S3. Importantly, large errors are also visible along the edges of moored buoy coverage (Fig. 2d and e) in OSSEs similar to the results of OSEs (Fig. 2a and b). It is also noticed that the shocks are generated not right on the boundaries but some distance away from the boundary. This may be because the observations on the boundaries assert their influence up to some distance determined by the localization radius and/or horizontal model error covariance thereby taking care of shocks right in the vicinity of the boundaries. For instance, the influence of observations decreases by a Gaussian taper with standard deviation of 6 degrees for observations at 10°S with SPEEDY-NEMO-LETKF, and 4 degrees for INCOIS-GODAS (Refer Fig. 1 of ref. 32 for more discussion on horizontal model error covariance structures). These results from OSEs and OSSEs are striking and indicate that the errors generated at the edges of moored buoy observations are generic in nature and not specific to the choice of a model or assimilation method. It is worth mentioning here that such errors also appear in the experiment where the moored buoy coverage is artificially increased from 10°S-10°N to 40°S-40°N demonstrating that the generation of errors at edges of a banded observation network is independent of the location of the confining latitude (refer to section 5 of supplementary information).

**Figure 3:**
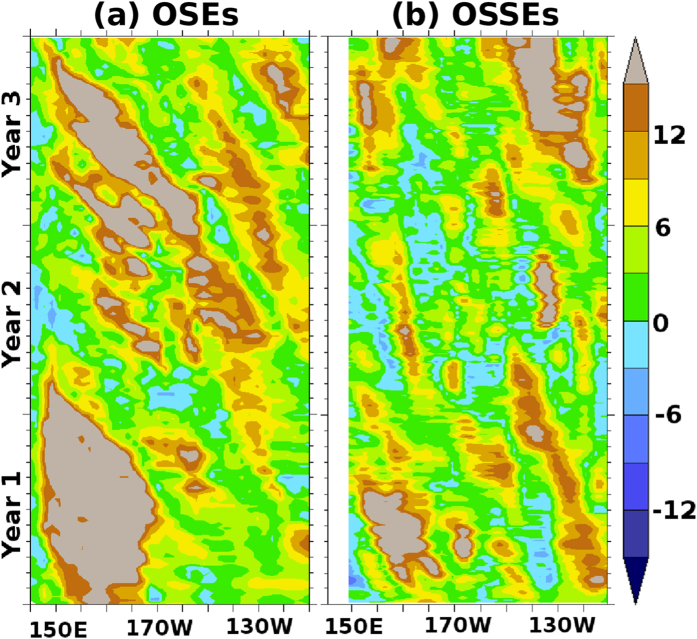
Hovmoller diagram of absolute error difference in SSHA (cm) between *MB* and *FR* experiments from (**a**) OSEs centered around 15°S and (**b**) OSSEs centered around 20°S. Positive (negative) values indicate degradation (improvements) from the *MB* with respect to *FR*. Results from the first three year simulations are plotted. Images are generated and processed with the help of FERRET-V6.3 (www.ferret.noaa.gov/Ferret) and GIMP-V2.8 (www.gimp.org) respectively.

Hovmoller diagrams for OSEs (Fig. 3a) and OSSEs (Fig. 3b) demonstrate that these errors, which we hence-forth call “assimilation shocks”, travel westward at a speed of around 10 *cm/sec* at 20°S which is very similar to oceanic Rossby wave speeds at these latitudes^33^. We believe that assimilation of moored observations produces repeated adjustments in and around the observation location during each assimilation cycle. Parts of these adjustments get annihilated for locations inside the boundary at the next analysis cycle and the rest dissipates following the model dispersion and dissipation. However, at locations outside the observational influence, the model dynamics are not sufficient to mitigate these shocks and they accrue over time leading to a spatial band-like structure of errors at the edge of the observation coverage. Westward propagation of these errors leads to further intensification near the western boundaries (Fig. 2a,b,d and e).

## Discussion

Earlier studies^26^,^34^,^35^,^36^,^37^,^38^ have shown the existence of significant degradations in one or two ocean state variables due to assimilation arising out of dynamical imbalances. Efforts were made to take care of such imbalances using balance operators and they met with some success. However, none of these studies pointed out the existence of large degradations and their subsequent propagation arising out of fixed observations in limited regions, like moored buoys. Unlike previous studies, we report degradations in the assimilated variables as well. Degradations along the edges may have been overlooked due to the remedy offered by Argo observations with which the shock is much mitigated. For example, in Fig. 2c and f we highlight the effect of Argo assimilation in addition to moored buoy assimilation. Figure 2f is a plot of difference in RMSE of SSHA of the *AR*_*S*_+*MB*_*S*_ and the *FR*_*S*_ run. It can be seen that the large spatial errors that were seen in Fig. 2e are mostly corrected by Argo observations. A similar result is observed in Fig. 2c, which plots the difference in RMSE of SSHA of the *AR*_*R*_+*MB*_*R*_and the *FR*_*R*_model run from OSEs. It is not surprising that Argo floats mitigate the errors at the moored-buoy boundaries because every now and then Argo floats pop up randomly at the boundaries and reduce these shocks. We note that assimilating Argo alone in OSSEs presents large improvements and no or minimal shocks are seen owing to homogeneous global coverage (figure not shown). In OSEs large improvements are seen within the Argo buoys assimilation region (60°*S*-60°*N*) and conversely, large errors emerge south of 60°*S* (figure not shown) reiterating the problem of error growth at the edges of a banded observational network.

**Figure 4:**
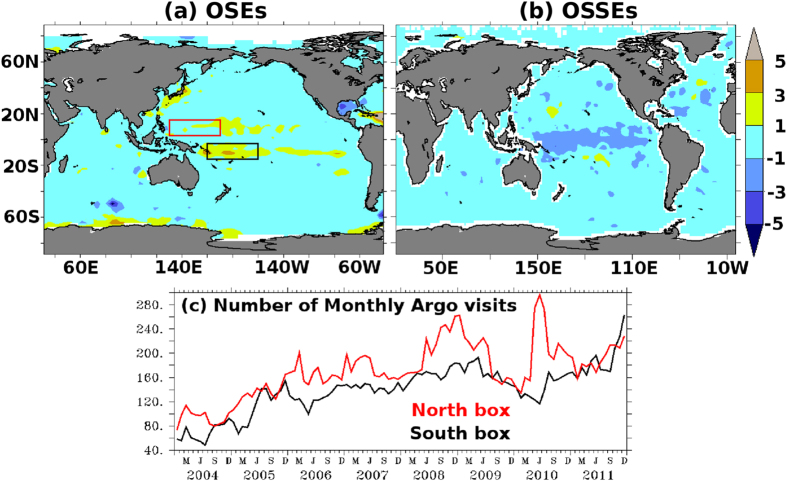
Root-mean-squared error differences in SSHA (cm) between *(MB*+*AR)* and *AR* in (**a**) OSEs and (**b**) OSSEs. Positive (negative) values indicate degradation (improvements) from the (*MB*+*AR)* with respect to *AR*. (**c**) Monthly time series of number of Argo visits in the North (red) and South (black) box. The geographical boundaries of North (130°*E*-170°*E* & 3°*N*-15°*N*) and South (160°*E*-160°*W* & 3°*S*-15°*S)* boxes are indicated in panel (a). Images are generated and processed with the help of FERRET-V6.3 (www.ferret.noaa.gov/Ferret) and GIMP-V2.8 (www.gimp.org) respectively.

In order to further verify whether addition of Argo to the moored buoy observations mitigates errors completely, in Fig. 4b (4a), we plot the improvement of (*AR*+*MB*)_*S(R*)_ with respect to *AR*_*S(R*)_ for OSSEs(OSEs). Results from these OSSEs show that the presence of Argo alongside moored buoys not only mitigates the shocks at the edges of moored buoys but also introduces a positive assimilation impact (Fig. 4b). For instance, large RMSE over 140°*E*-110°*W* & 18°*S*-22°*S* region - the region where large assimilation shocks are noticed in *MB*_*S*_ experiment of OSSEs - is improved from 6.9 *cm* in *MB*_*S*_to 2.0 *cm* in *AR*_*S*_+*MB*_*S*_. These improvements in *AR*_*S*_+*MB*_*S*_are better than *AR*_*S*_ alone, for which RMSE of 2.3 *cm* is observed. In other words, Argo floats and moored buoys complement each other in the OSSEs. On the other hand, results from OSEs are slightly different. Addition of Argo to moored buoy observations could not completely eliminate the assimilation shocks at the edges of moored buoy (Fig. 4a). For example, large RMSEs of *MB*_*R*_ are reduced from 11.0 *cm* to 3.5 *cm* after adding Argo to it over 160°*E*-160°*W* & 3°*S*-15°*S* region where large assimilation shocks are noticed in *MB*_*R*_ of OSEs. However, these RMSEs are still larger than those observed in *FR*_*R*_ (3.0 *cm*). They are reduced further (2.2 *cm*) when only Argo is assimilated, i.e., no moored buoy assimilation (*AR*_*R*_). It is reasonable to speculate that the persistence of errors in *AR*_*R*_+*MB*_*R*_ is due to insufficient Argo coverage to counter the assimilation shocks at the edges of moored buoy. For instance, North-Western region of the tropical Pacific (red box in Fig. 4a covering 130°*E*-170°*E* & 3°*N*-15°*N*), where no or very little degradations appear, experienced about 180 Argo visits per month on an average. By contrast, the South-Western region (black box in Fig. 4a covering 160°*E*-160°*W*& 3°*S*-15°*S*) of the tropical Pacific, where largest of degradations appear, has always experienced fewer Argo visits than the northern region (Fig. 4c).

**Figure 5:**
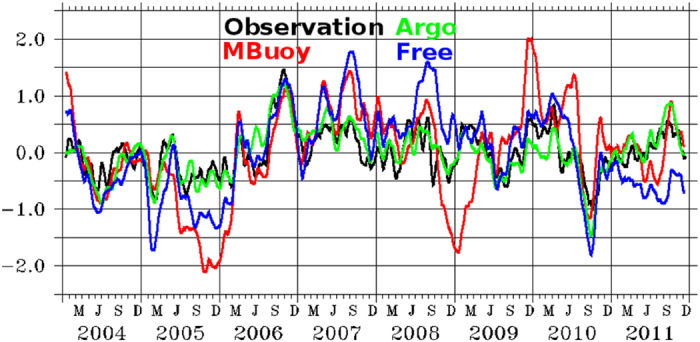
Indian Ocean Dipole mode (°C) estimated from the observation (black) from TMIAMSRE-SST and simulations from *MB*_*R*_ (red), *AR*_*R*_ (green) and *FR*_*R*_ (blue) experiments. Image is generated and processed with the help of FERRET-V6.3 (www.ferret.noaa.gov/Ferret) and GIMP-V2.8 (www.gimp.org) respectively.

While assimilation shocks during Argo-era (2004 onwards) have little or no signature on the ocean re-analysis thanks to near homogeneous global Argo coverage, assimilation shocks might have crept inside ocean re-analysis during the pre-Argo era owing to the banded observation coverage. Here we illustrate a possible erroneous inference that could have happened in the absence of Argo by analyzing Indian Ocean Dipole (IOD) mode index which is defined^39^ as the difference between SST anomalies of tropical western Indian Ocean (50°*E*-70°*E*, 10°*S*-10°*N*) and tropical south-eastern part of the Indian Ocean (90°*E*-110°*E*, 10°*S*-*Equator*). It is known that IOD explains 12% of the SST variability in the Indian Ocean region^39^ and has significant influence on the global climate especially the Indian Ocean rim countries^40^. Figure 5 shows IOD index derived from the observation and model simulations of *MB*_*R*_ and *AR*_*R*_. It can be observed that while *AR*_*R*_has excellent agreement with the observation thanks to homogeneous Argo coverage in the Indian Ocean, *MB*_*R*_ experiment wrongly simulates 2005 and 2008 as strong negative IOD years. It is worth mentioning here that these discrepancies are larger than the *FR*_*R*_ even between 2007–2011 which boasts of improved moored buoy coverage in the Indian Ocean. This result clearly questions the fidelity of ocean re-analysis in the pre-Argo era. The observations from tropical moorings in the Pacific are used in the ocean re-analysis since 1985 and the global Argo program was implemented only in 1999 and reached the global coverage goal of 3000 floats in 2007^19^. Hence it presents possibilities for potential inaccuracies in global ocean re-analysis before about 2000. Since it has been demonstrated that SSHA assimilation does not improve upon temperature and salinity^35^,^41^ to the same extent as Argo does, we believe that there may be shocks if SSHA is assimilated in the pre-Argo era thereby leading to possible incorrect inferences about climate change studies. Similar spurious waves might propagate in atmospheric systems as well but they are likely to disperse or dissipate quickly owing to the faster timescales of the atmospheric dynamics, a hypothesis that we plan to test.

In summary, assimilation systems in the ocean generate shocks at the edges of observation bands. These shocks accrue over time and also propagate westward as Rossby waves. Since these shocks are present in different assimilation systems, we believe it to be a fundamental deficiency in assimilation of observations at fixed latitudinal bands. Though global coverage of Argo provides respite from these errors in the ocean re-analysis, care must be taken before interpreting ocean re-analysis especially before Argo era.

## Additional Information

**How to cite this article**: Sivareddy, S. *et al*. The pre-Argo ocean reanalyses may be seriously affected by the spatial coverage of moored buoys. *Sci. Rep.* **7**, 46685; doi: 10.1038/srep46685 (2017).

**Publisher's note:** Springer Nature remains neutral with regard to jurisdictional claims in published maps and institutional affiliations.

## Supplementary Material

Supplementary Information
