# Supplementary material for: The pre-Argo ocean reanalyses may be seriously affected by the spatial coverage of moored buoys
Source: Sci Rep. 2017 Apr 21;7:46685. doi: 10.1038/srep46685 (PMC5399374; doi:10.1038/srep46685)
Supplement: Supplementary Information [file srep46685-s1.doc]

Supplementary Information

**The pre-Argo ocean reanalyses may be seriously affected by the spatial coverage of moored buoys**

S. Sivareddy^1^,Arya Paul^1^, Travis Sluka^2^, M. Ravichandran^1&3^, and Eugenia Kalnay^2^

*1. ESSO-Indian National Centre for Ocean Information Services, Ministry of Earth Sciences, Pragathi Nagar, Hyderabad-500090, India*

*2. Department of Atmospheric and Oceanic Sciences, University of Maryland, College Park, Maryland, USA*

*and*

*3.ESSO-National Centre for Antarctic and Ocean Research, Ministry of Earth Sciences,*

*Headland Sada, Vasco-da-Gama, Goa 403804, India*

**S1. Description of INCOIS-GODAS**

INCOIS-GODAS consists of an Ocean General Circulation Model (OGCM), the Modular Ocean Model (MOM) version 4.0, coupled to 3D-Var assimilation scheme adopted from the assimilation system developed by National Centre for Environmental Prediction (referred to as NCEP-GODAS^1^). The OGCM has uniform zonal resolution of 0*.*5°. The meridional resolution is 0*.*25° till 10° from the equator and decreases exponentially from 10°*N* (10°*S*) to 30°*N* (30°*S*) so that 0*.*5° uniform resolution is maintained north(south) of 30°*N* (30°*S*). There are 40 layers in the vertical direction with the top 24 layers placed every 10m. Model integration time step is 30 minutes. The model is forced with NCEP-R2 atmospheric fluxes^2^. The 3D-VAR assimilation scheme implemented in INCOIS-GODAS assimilates observed *in-situ* temperature and salinity profiles within 60°*S*-60°*N* and from surface to 750m depth. The assimilation is performed every 6 hrs. For the assimilation, observations during -10 days to +10 days of the assimilation cycle are used. Further details about the performance of INCOIS-GODAS under various configurations can be found in^3^. An inter-comparison performed between ocean re-analysis of INCOIS-GODAS, NCEP-GODAS and ECMWF-ORAS4 (discussed in chapter 6 of [3]) shows that the INCOIS-GODAS is suitable for conducting OSEs in Indian and Pacific Ocean. In the present study, we use the same configuration of INCOIS-GODAS as that discussed in [3] except for sea surface temperature (SST) relaxation. Turning off SST relaxation for the present exercise offers an advantage to have an independent source of SST for comparisons while assessing the impact. The results do not change even if SST is relaxed (figures not shown).

**S2 Description of the SPEEDY-NEMO-LETKF system experiments under OSSE**

Different configurations of SPEEDY-NEMO-LETKF used under OSSEs are described below.

**Nature Run**

The nature run, which reflects the truth of the system, is performed using simple coupled ocean-atmospheric model SPEEDY-NEMO^4, 5^. The atmospheric general circulation model, Simplified Parameterizations PrimitivE-Equation DYnamics (SPEEDY)^5^, is coupled to the OGCM, Nucleus for European Model of the Ocean (NEMO)^6^ every 6hrs. SPEEDY is configured using 8 layers in the vertical and a triangular spectral truncation at total wave number 30 (T30) in the horizontal direction. Integration time step for SPEEDY is set at 40 minutes. NEMO uses orthogonal curvilinear grid in the horizontal and z-coordinates in the vertical. It is configured with quasi-global set up covering 78°*S-* 90°*N* with variable horizontal resolution of 2° *X* 0*.5°* around Equator and around 70°*S*, and 2° *X* 2° in other regions of the ocean with integration time step of 90 minutes. It has 31 vertical levels with the resolution of 10*m* within the upper 100*m* and 500*m* near bottom. The SPEEDY-NEMO is evolved for 48 years initialized from climatological temperature and salinity in the ocean. The first 20 years are used for the system spin-up^7^ while during the last 22 years, NEMO is relaxed to SST and SSS monthly Levitus climatologies^8^. We consider the last 22 years of the ocean state as the truth of the system. This 22 year *Nature* experiment (truth) also forms the source to sample simulated temperature and salinity observations in the ocean.

**The imperfect free model**

We introduce imperfections in the model through the initial conditions. We generate a 32-member ensemble of imperfect systems by turning off sea surface temperature and sea surface salinity relaxations in the ocean model and randomly picking initial conditions from all January months of 22-year *Nature* run. This assimilation free 32-ensemble coupled system is then evolved for three years under *FR_S_* experiment. In Fig.S1a, we plot the mean and individual net heat flux the ocean surface (averaged over 1*50°E*-160°*W* & 20°*N*-35°*N*) of all the 32 members and compare it against the truth. The ensemble mean of the net heat flux is slightly different from the truth as intended. Also, ensemble spread is large enough to contain the truth within it. These qualities demonstrate that the *FR_S_* experiment is appropriate to be used in the OSSEs.

**The Assimilation System**

The assimilation scheme Local Ensemble Kalman Filter (LETKF)^9^, developed for large spatio-temporally chaotic systems such as atmosphere and oceans, is coupled to the imperfect free model, NEMO to form an assimilation system similar to SPEEDY-NEMO-LETKF^7^. Our assimilation system differs from [7] in the following sense – (1) atmospheric assimilation is turned off, (2) 32 instead of 40 ensemble members are used in the assimilation enabled experiments, and (3) simulated temperature and salinity observations are assimilated every 24 hours instead of 6 hours. In addition, and most importantly, (4) the atmospheric component SPEEDY is decoupled from NEMO. The imperfect free ocean model, NEMO used here is forced by the atmospheric fluxes derived in the *FR_S_* experiment. The same *FR_S_* oceanic initial conditions are used to initiate ocean model ensemble members. Hence, comparison of ocean states between the free (*FR_S_*) and the assimilating systems (e.g., the *MB_S_* experiment assimilating moored buoys) should describe well the impacts of assimilation. A variable localization radius of 720*km* near equator and 200*km* at the poles is used during assimilation. In order to account for the decrease in ensemble spread due to assimilation analysis spread is relaxed to guess spread using a relaxation parameter^10^. The relaxation parameter is set to 0.98.


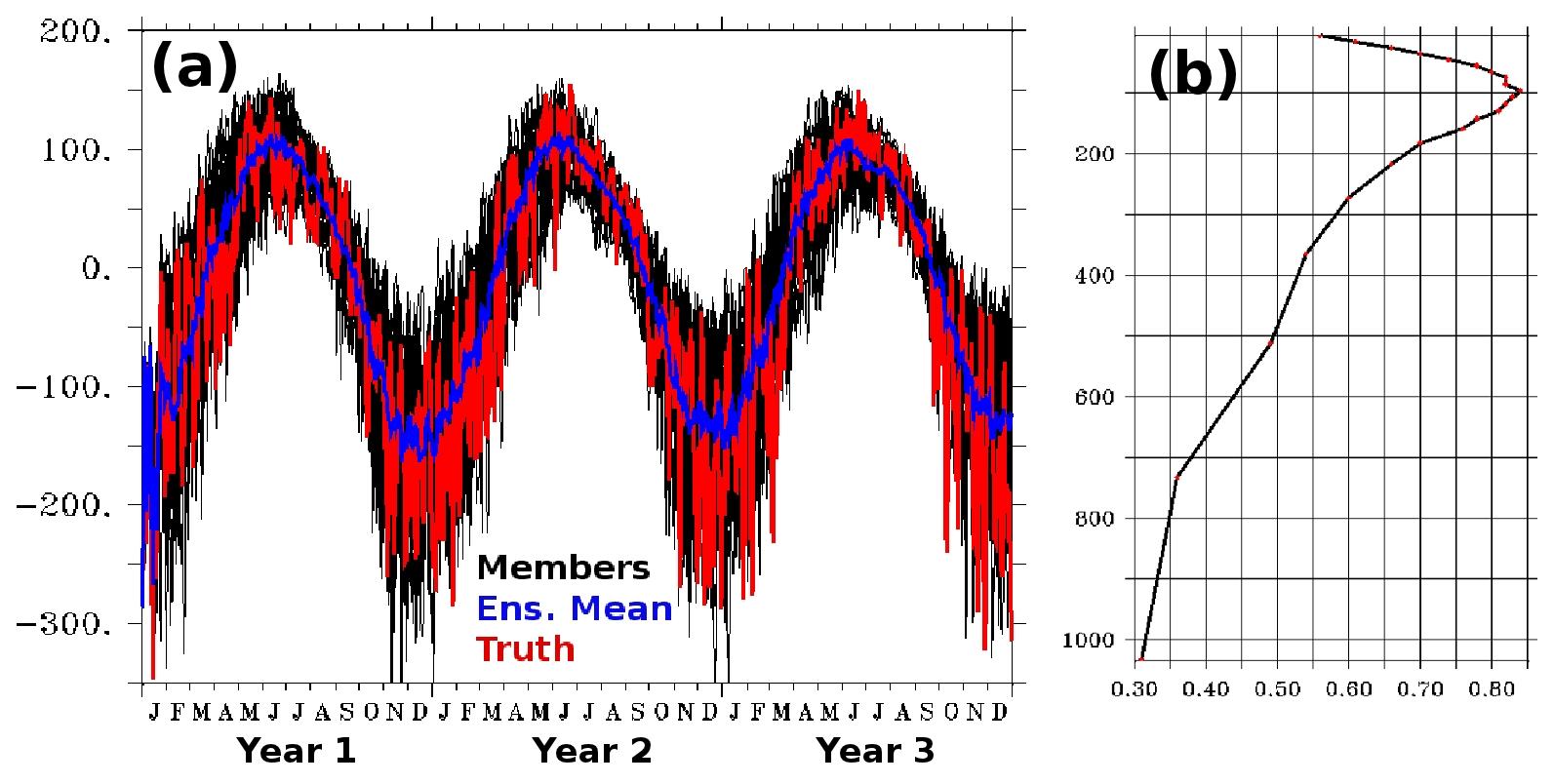


**Figure S1.** (a) Net heat flux at the ocean surface averaged over 150°*E*-160°*W*& 20°*N*-35°*N* region from *Nature* experiment (red solid line) and all 32-member ensembles of *FR_S_* experiment (thin black lines). The Figure also shows mean of the ensemble (solid blue line). Units are in *W m*^-2^. (b) Depth-wise profile structure of errors assigned to temperature (expressed in °*C*) and salinity (expressed in *psu* multiplied by 10) observations while sampling from the truth. Red dots indicate ocean model grid points in the vertical direction. Images are generated and processed with the help of FERRET-V6.3 (www.ferret.noaa.gov/Ferret) and GIMP-V2.8 (www.gimp.org) respectively.

The simulated observations generated from the Nature run are assimilated into the NEMO ocean model. Temperature and salinity observations are simulated by adding a Gaussian white noise having a depth-dependent standard deviation to the ocean state obtained in the Nature Run. This presents us with extensive control over simulations of observations. We have the liberty to specify spatio-temporal configuration of observations and also to bestow it with intended statistical properties. The depth-dependent standard deviation used for simulating temperature and salinity observations has its maximum (0*.*84°*C* for temperature and 0.084*psu* for salinity*)* at 100m to account for large variabilities (and hence large representative errors) close to the thermocline (Fig. S1b). It is to be noted here that temperature and salinity observations are sampled only on model grid points. This is done to avoid interpolation errors.

**S3. Demonstration of improvements within assimilation region in both INCOIS-GODAS and SPEEDY-NEMO-LETKF**

Fig. S2 illustrates the ability of the data assimilation system in both OSEs and OSSEs. Fig. S2d, S2e, S2f highlight the improvements with respect to *Nature* run of SST, SSS and sea-surface height (SSH) averaged over Central Pacific (140°*E*-170°*W*& 5°*S*-5°*N*) due to assimilation of moored buoy observations in the *MB_S_* of OSSE experiment during the first three years of simulations. Figs. S2a, S2b, S2c show similar improvements in the OSEs during the period 2004-2011. The reference for SST in OSEs is TMIAMSRE^24^ (a level-4 product based on SST observations of TMI and AMSRE satellites) during that period whereas for SSS and SSH, we consider the results of a system wherein all types of *in-situ* temperature and salinity observations are assimilated as reference owing to non-availability of SSS and SSH observations during that period. All these figures show that the system - post assimilation - can simulate the variabilities present in the "truth" which were earlier absent in the non-assimilated system.


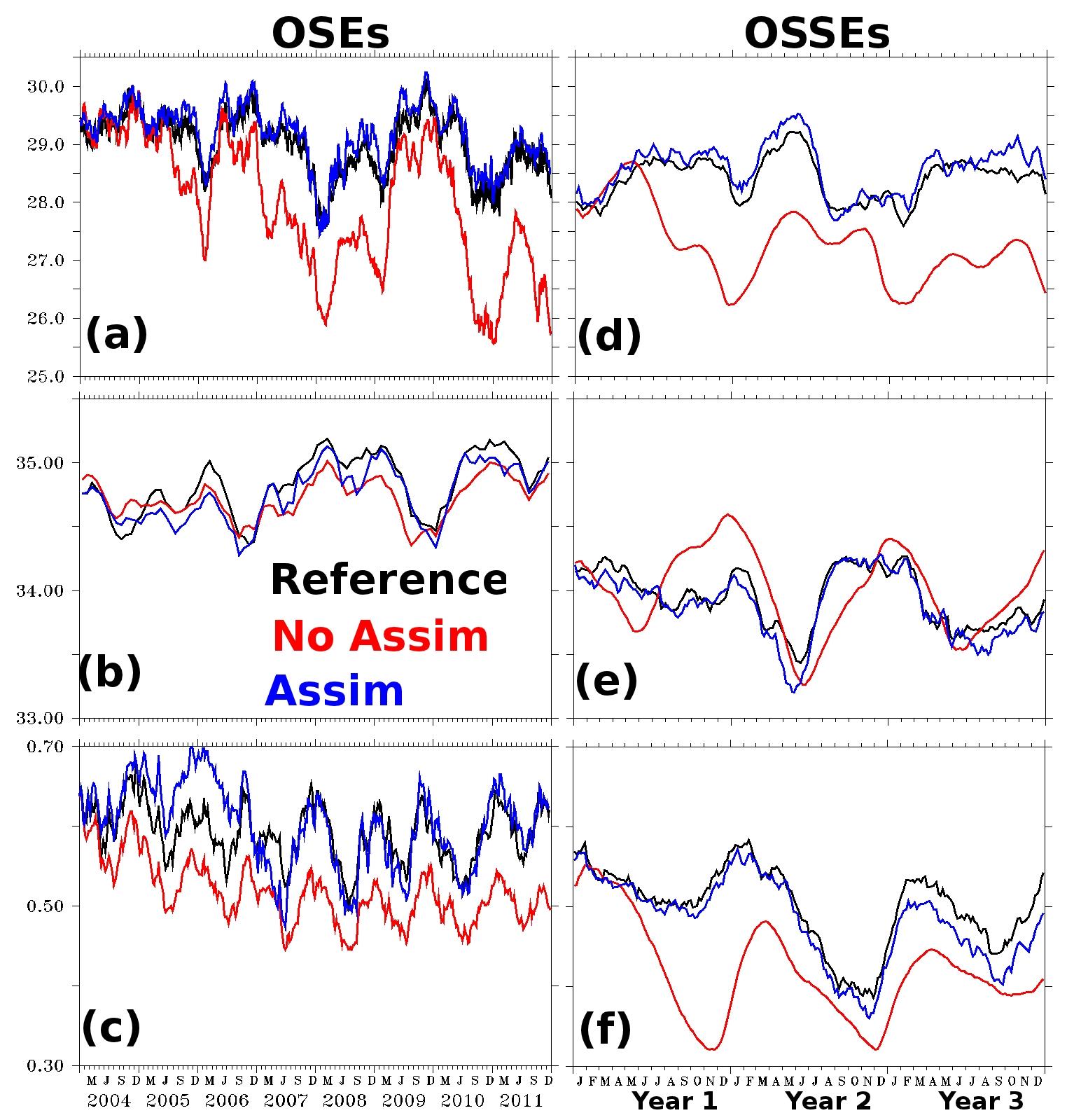


**Figure S2.** Sea Surface Temperature (SST; °*C*; a &d), Sea Surface Salinity (SSS; *psu*; b&e) and Sea Surface Height (SSH; *cm*; c& f) averaged over 150°*E*-160°*W*& 5°*S*-5°*N* region from *Reference* (black), *FR_R_* (red), and *MB_R_* (blue) experiments in OSEs (a, b, &c). Similar plots are shown for OSSEs in d, e, &f corresponding to *Reference* (black), *FR_S_* (red), and *MB_S_* (blue) experiments. *Reference* in OSSEs is *Nature* experiment. Whereas in OSEs *Reference* is the *TMIAMSRE* for SST comparisons, and for SSS and SSH it is the *REF* experiment wherein *in-situ* observations from all sources are used for assimilation. Images are generated and processed with the help of FERRET-V6.3 (www.ferret.noaa.gov/Ferret) and GIMP-V2.8 (www.gimp.org) respectively.

**S4. Discussion on the contrasting SSHA results between OSEs and OSSEs within moored buoy coverage area.**

In Figure S2, we plot the mean SSH of FR, MB and Reference Experiment of both (c) OSEs and (f) OSSEs. In both OSEs and OSSEs*,* the mean SSH simulated by FR inside the moored buoy coverage area suffers from a significant bias. Assimilating moored-buoy observations improves the mean SSH in both OSEs and OSSEs. Unlike the OSE simulations, FR_S_ in OSSEs fails to capture the variabilities, especially at intra-seasonal scales, in mean SSH inside the boundaries (refer Figure S2f and Figure S3b). However, assimilation of moored buoys helps in capturing variabilities inside the coverage area in *MB_S_*.  It is to be kept in mind that assimilating observations generate imbalances unless explicitly taken care of. These imbalances trigger transient waves which in most of the cases either die down before the next analysis cycle or is mitigated by the assimilation of observations in the next analysis cycle. In *MB_S_*, the improvements due to assimilation in the variabilities especially at intra-seasonal frequencies overwhelm small modulations due to these transient waves. Whereas in OSEs,  since *FR_R_* had no problems in capturing variabilities (refer Figure S2c and S3a), the scope of improvements in SSHA is minimal and these modulations due to transient waves dominate over the minimal improvements thereby effectively degrading the analysis inside the boundaries.


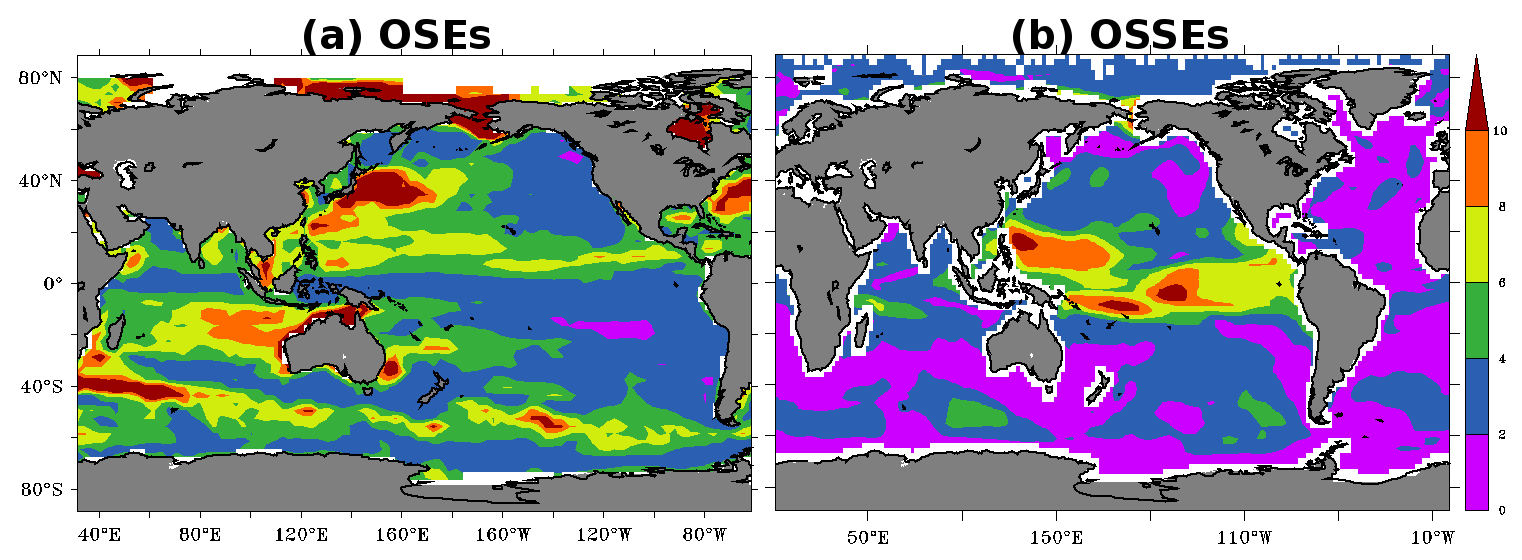


**Figure S3.** RMSE in SSHA (cm) in (a) *FR_R_* and (b) *FR_S_*. RMSE is estimated using altimeter SSHA observations and SSHA derived from Nature run for *FR_R_* and *FR_S_* respectively. Statistics are based on simulations of 2005-2011 and last 2 years in *FR_R_* and *FR_S_* respectively. Images are generated and processed with the help of FERRET-V6.3 (www.ferret.noaa.gov/Ferret) and GIMP-V2.8 (www.gimp.org) respectively.

**S5. Demonstration of genesis of assimilation shocks for different latitudes**

We performed an experiment under OSSE where we extended the moored buoy coverage from 10°S-10°N to 40°S-40°N in order to understand the effect of extended moored buoy coverage on assimilation shocks. We still observe shocks at the edges of the moored buoy boundaries (Fig. S4).


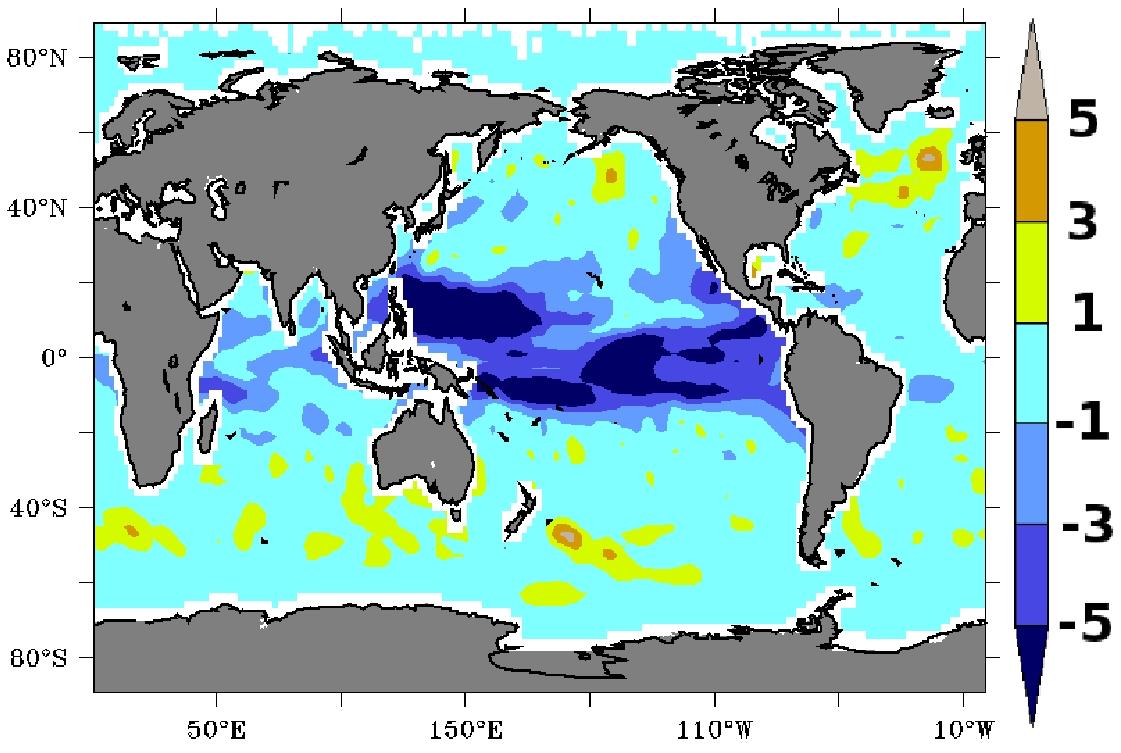


**Figure S4.** Root-mean-squared-error differences in SSHA (cm) between *FR_S_*and*40°S-40°N* (the experiment where moored buoy like observations are assimilated between 40*°S* and 40*°N*). Root-mean-squared-error for each experiment is computed with respect to *Nature* run. In the figure positive (negative) values indicate degradation (improvements) from the assimilation experiment with respect to free experiment. Image is generated and processed with the help of FERRET-V6.3 (www.ferret.noaa.gov/Ferret) and GIMP-V2.8 (www.gimp.org) respectively.

**References**

1. Behringer, D.W. & Xue, Y. Evaluation of the global ocean data assimilation system at NCEP: The Pacific Ocean. In *Proc. Eighth Symp.on Integrated Observing and Assimilation Systems for Atmosphere, Oceans, and Land Surface*. (2004)
2. Kanamitsu, M., Ebisuzaki, W., Woollen, J. & Shi-Keng, Y. Ncep-doe amip-ii reanalysis (r-2). *Bulletin of the American Meteorological Society*. **83(11)**, p.1631 (2002).
3. Sivareddy, S. A study on global ocean analysis from an ocean data assimilation system and its sensitivity to observations and forcing fields, Ph.D. thesis, Andhra University. Available at <http://www.incois.gov.in/documents/PhDThesis_Sivareddy.pdf>. (2015)
4. Kucharski, F. et al. Atlantic forcing of Pacific decadal variability.*Climate Dynamics*. **46(7-8)**, pp.2337-2351 (2016).
5. Kucharski, F., Molteni, F., King, M.P., Farneti, R., Kang, I.S. & Feudale, L. On the need of intermediate complexity general circulation models: a “SPEEDY” example. *Bulletin of the American Meteorological Society*. **94(1)**, pp.25-30 (2013).
6. Madec, G. Nemo ocean engine in *Note du Pole de modlisation, Institut Pierre-Simon Laplace.* (2008)*.*
7. Sluka, T.C., Penny, S.G., Kalnay, E. & Miyoshi, T. Assimilating atmospheric observations into the ocean using strongly coupled ensemble data assimilation. *Geophysical Research Letters*. ***43*(2)**, pp.752-759 (2016).
8. Conkright, M., Levitus, S., O’Brien, T., Boyer, T., Antonov, J. & Stephens, C. World Ocean Atlas 1998 CD-ROM data set documentation. *National Oceanographic Data Center (NODC) Internal Report, Silver Spring, Maryland. (1998)*.
9. Hunt, B.R., Kostelich, E.J. & Szunyogh, I. Efficient data assimilation for spatiotemporal chaos: A local ensemble transform Kalman filter. *Physica D: Nonlinear Phenomena*. **230(1)**, pp.112-126 (2007).
10. Whitaker, J.S. & Hamill, T.M. Evaluating methods to account for system errors in ensemble data assimilation. *Monthly Weather Review*. **140(9)**, pp.3078-3089 (2012).
